# Not so different after all? An event-related potential study on item and source memory for object-scene pairs in German and Chinese young adults

**DOI:** 10.3389/fnhum.2023.1233594

**Published:** 2023-09-13

**Authors:** Michael Weigl, Qi Shao, Enno Wang, Zhiwei Zheng, Juan Li, Jutta Kray, Axel Mecklinger

**Affiliations:** ^1^Department of Psychology, Saarland University, Saarbrücken, Germany; ^2^Center on Aging Psychology, CAS Key Laboratory of Mental Health, Institute of Psychology Chinese Academy of Sciences, Beijing, China; ^3^Department of Psychology, University of Chinese Academy of Sciences, Beijing, China

**Keywords:** culture, item memory, source memory, ERP, old/new effect

## Abstract

In recent years, several cross-cultural studies reported that Westerners focus more on central aspects of a scene (e.g., an object) relative to peripheral aspects (e.g., the background), whereas Easterners more evenly allocate attention to central and peripheral aspects. In memory tasks, Easterners exhibit worse recognition for the central object when peripheral aspects are changed, whereas Westerners are less affected by peripheral changes. However, most of these studies rely on hit rates without correcting for response bias, whereas studies accounting for response bias failed to replicate cultural differences in memory tasks. In this event-related potential (ERP) study, we investigated item and source memory for semantically unrelated object-scene pairs in German and Chinese young adults using memory measures corrected for response bias (i.e., the discrimination index Pr). Both groups completed study-test cycles with either item memory tests or source memory tests. In item memory blocks, participants completed an old/new recognition test for the central object. Source memory blocks entailed an associative recognition test for the association between object and background. Item and source memory were better for intact than for recombined pairs. However, as verified with frequentist and Bayesian analyzes, this context effect was not modulated by culture. The ERP results revealed an old/new effect for the item memory task in both groups which was again not modulated by culture. Our findings suggest that cultural differences in young adults do not manifest in intentional memory tasks probing memory for object-scene pairs without semantic relations when using bias-corrected memory measures.

## 1. Introduction

Studies on neural plasticity found that becoming a London taxi driver (Maguire et al., 2000) or learning to juggle (Draganski et al., 2004) can alter the brain even on a macrostructural level. In a similar manner, life-long exposure to a specific culture can sculpt not only social behavior, but also lower-level perceptual and cognitive functions and their associated neural correlates (Nisbett, 2003; Park and Gutchess, 2006; Gutchess and Huff, 2016). Cultural differences have been observed for perceptual and cognitive processes such as causal attribution, categorization, scene perception, attention allocation, and memory (Nisbett, 2003; Nisbett and Masuda, 2003; Masuda, 2017). In this paper, we will focus on the last two processes, namely attention allocation and memory.

In their seminal study, Masuda and Nisbett (2001) proposed that cultural differences in attention allocation and memory can be attributed to differences between an analytic and a holistic thinking style. Westerners (typically referring to people from North America and Europe) exhibit an analytic thinking style, which is characterized by an object-oriented attentional focus. In other words, Westerners tend to focus more on central aspects of a scene at the expense of the periphery. By contrast, East Asians (typically referring to people from China, Korea, and Japan) prefer a holistic thinking style, which is characterized by a context-and relation-oriented attentional focus (Masuda and Nisbett, 2001; Nisbett and Masuda, 2003). In two experiments, Masuda and Nisbett (2001) presented their American and Japanese participants object-background arrangements (e.g., various fishes in an underwater scene, animals in different environment). They found that Americans allocated their attention on the foreground object, whereas the Japanese participants also focused on the surrounding objects and background. These differences in attention allocation had mnemonic consequences. In a subsequent memory task, both groups were exposed to intact and recombined object-background arrangements (intact: object presented on the same background as in the study phase; recombined: object presented on a different background as in the study phase). Japanese exhibited worse recognition for the central object when peripheral aspects were changed (i.e., for recombined items), whereas Americans were less affected by peripheral changes.

Subsequently, studies using eye-tracking (Chua et al., 2005; Masuda et al., 2008) or functional magnetic resonance imaging (fMRI; Gutchess et al., 2006) provided more evidence for the existence of cultural differences in attention allocation and visual processing between Westerners and East Asians, which were consistent with the notion of analytic vs. holistic processing (see Masuda, 2017, for a review). In addition, many studies (Chua et al., 2005; Masuda et al., 2008; Evans et al., 2009; Ko et al., 2011; Mickley Steinmetz et al., 2018) used a similar paradigm as Masuda and Nisbett (2001) to investigate cultural differences due to differences in analytic vs. holistic processing. Participants from Western and East Asian cultures studied pictures of objects or persons in front of a background scene and later had to remember central stimulus aspects, peripheral stimulus aspects, or both. Typically, recognition memory for the central object is more hampered by peripheral information in East Asians than in Westerners (e.g., Masuda and Nisbett, 2001; Chua et al., 2005; Masuda et al., 2008; Mickley Steinmetz et al., 2018). There is also evidence that Westerners are better at distinguishing studied objects from similar, but unstudied ones (i.e., lures), suggesting that memory specificity is higher in Westerners as compared to East Asians (Millar et al., 2013; Leger and Gutchess, 2021). At the same time, East Asians showed superior memory for background information relative to Westerners (e.g., Ko et al., 2011). Together, these results support the view that East Asians adapt a more holistic and Westerners a more analytic processing style. Moreover, the cultural difference in processing style results in mnemonic consequences.

However, a couple of studies challenge the view of cultural differences in memory. Most of the aforementioned studies rely on hit rates without correcting for response bias. This is problematic, because the cultural groups could have similar memory performance and might differ only in their decision criterion (i.e., response bias). Hypothetically speaking, the East Asian and Western participants in those studies could have had similar recognition memory for old object on new backgrounds. Yet, the East Asian participants might have responded “old” only if they were highly confident. In fact, some studies accounting for response bias failed to replicate cultural differences in memory tasks (Gutchess and Huff, 2016). For example, a study which combined eye-tracking with receiver operator characteristic analysis, which simultaneously considers hit rates and false alarm rates at different levels of confidence in recognition memory judgments (and thereby account for response bias Yonelinas and Parks, 2007), failed to find any cultural differences in memory or eye fixations (Evans et al., 2009). Relying on hit rates alone is also problematic on a conceptual level. For example, Masuda and Nisbett (2001) make claims about the association between foreground object and background scene (i.e., about associative memory). However, by using hit rates they only test for the accuracy of recognizing the object (i.e., for item memory).

Another reason for these replication failures could be found in variations of the stimuli. Masuda and Nisbett (2001) used animals placed in front of a background scene in their study. Most studies replicating the cultural differences in memory relied on similar stimuli (e.g., Chua et al., 2005; Masuda et al., 2008), whereas studies failing to find cultural differences used different materials [e.g., identity of the speaker of a piece of information as in Chua et al., 2006]. For example, Chua et al. (2006) presented younger and older Americans and Chinese statements spoken by different speakers. Contrary to what would be expected from the higher context sensitivity of East Asians, they did not observe any cultural differences in memory for the speakers. In the study of Yang et al. (2013), younger and older Canadians actually had superior source memory than their Chinese counterparts.

Thus, whether culture affects episodic memory remains uncertain, and importantly the exact neurocognitive mechanisms by which culture affects episodic memory are still understudied. Here, we investigated younger adults’ memories with both behavioral and electrophysiological measures, which tend to be more sensitive than behavioral measures to disclose subtle cultural differences in memory. In more detail, we investigated item and source memory for objects presented in front of studied and unstudied background scenes while recording EEG activity, which was used to analyze event-related potentials (ERP).

ERPs have many advantages over other neurophysiological methods. The ERP technique is inexpensive, easy to apply, and non-invasive (e.g., Luck, 2005). Furthermore, they have a high temporal resolution, which allows researcher to observe cognitive processes as they unfold with minimal delay (Luck, 2005).

A large amount of research has identified several ERP components related to episodic memory, in particular, to recognition memory (see Rugg and Curran, 2007, for a short review). Dual-process models of recognition memory distinguish between a fast and strength-based process called familiarity and a slower, threshold-driven process called recollection (Yonelinas, 2002). Furthermore, each of the two processes is associated with a distinct ERP component, namely the early and late old/new effect (Rugg and Curran, 2007, but see Paller et al. (2007), for a different view). The early old/new effect, also known as FN400, is correlated with familiarity-based retrieval and is typically observed between 300 and 500 ms with a mid-frontal scalp distribution. By contrast, the late old/new effect, sometimes also referred to as the late positive component (LPC), typically can be observed between 500 and 800 ms, has a left-parietal scalp distribution, and is correlated with recollection. Furthermore, the late old/new effect is also related to the retrieval of associative information (such as sources or context). The retrieval of unitized associations, however, is familiarity-based (Haskins et al., 2008) and has been linked to the early old/new effect (Bader et al., 2010; Kamp et al., 2016; Huffer et al., 2022, see Mecklinger and Bader, 2020, for a review). ERP memory effects can be observed even beyond the aforementioned time windows. Many ERP studies on memory reported the presence of a late posterior negativity (LPN) after 800 ms after stimulus onset, which is associated with reconstructive memory processes and the continued evaluation of retrieval outcomes (Leynes and Nagovsky, 2016; Li and Nie, 2021; Nie et al., 2023; see Mecklinger et al., 2016, for a review).

In this ERP study, we aimed at investigating cultural differences in item and source memory for semantically unrelated object-scene pairs in German and Chinese young adults. The stimuli were semantically unrelated object-scene pairs from the ORCA picture database (Weigl et al., 2023), which were arranged in a similar manner as in Masuda and Nisbett (2001). The ORCA picture database contains predefined intact and recombined object-scene pairs arranged in quadruples, which were already validated for cross-cultural research in a previous study (Weigl et al., 2023). In comparison with previous research, our material has the following advantages: (1) all object-scene compositions have visually and semantically matched distractors for every object and scene, (2) the selected stimuli have all a low semantic fit between the objects and scenes, and thereby preclude the influence of pre-existing associations, (3) all stimuli were rated by the target demographic (i.e., young German and Chinese adults), (4) all objects are equally familiar to both cultures, and (5) there is a large number of stimuli with a centered object, which reduces the necessity of eye-movements during EEG recording (see Weigl et al., 2023, for more details). As another advantage compared to prior studies, we employed memory measures corrected for response bias (i.e., the discrimination index Pr; Snodgrass and Corwin, 1988). In order to ensure comparability with the existing literature, accuracies (i.e., uncorrected hit rates) were also analyzed.

Based on the studies on cultural differences in item memory (e.g., Masuda and Nisbett, 2001; Chua et al., 2005), we predicted that Chinese participants should show worse item memory performance than German participants for old objects in new contexts, but item memory performance should be similar for Chinese and German participants for old items in the old context. In addition, we predicted that source memory for old objects on the original background should be better for Chinese relative to German participants, whereas source memory for old objects on a different background should be worse for Chinese relative to German participants.

For the ERPs, we predicted that Chinese participants would show a higher FN400 and a lower LPC (early and late old/new effects, respectively) relative to Germans due to unitization of object and background and, consequently, associative recognition driven by familiarity (e.g., Bader et al., 2010; Huffer et al., 2022). In a similar vein, we predicted that the FN400 would be lower for recombined items relative to intact items for Chinese participants as compared to German participants in the item memory test. Additionally, we explored whether the LPN was also sensitive to cultural differences in the processing of intact and recombined items.

## 2. Methods

### 2.1. Participants

Twenty-five students of the Saarland University in Saarbrücken, Germany ranging in age between 18 to 30 years and 32 students of the Chinese Academy of Science in Beijing, China ranging in age between 18 to 30 years (13 additional participants were excluded due to excessive EEG artifacts, see section 2.5 for more details). Sample size was determined via power analysis for the smallest effect size of interest for the memory tests (*f* = 0.40, α = 0.05, power (1−β) = 0.80, numerator df = 1, groups = 2). All subjects were native speakers of their country’s language (i.e., German/Chinese), had normal or corrected-to-normal vision and reported good health with no history of neurological or psychiatric illness. All participants gave written informed consent. The experimental procedures were approved by the Ethics Committee of the Institute of Psychology, Chinese Academy of Sciences and the Ethics Committee of the Faculty of Human and Business Sciences at Saarland University.

### 2.2. General procedure

The experimental session took around 3–3.5 h (including preparation for EEG). An overview over the structure of each session and the approximate duration for each step can be found in Table 1. Each session had the following structure.

**Table 1:** Procedure of the experiment.

Task order	Approx. duration
Pen test	2–3 min
Corsi-block	8–10 min
Medical & cultural screening	3–5 min
Edinburgh handedness inventory	1–2 min
Self-construal scale	3–5 min
Instruction and practice	20–25 min
Main experiment (EEG)	40–45 min
Decorum experiment (EEG)	10 min
Wechsler vocabulary test	10–15 min
Post-experimental questionnaire	5 min

At the beginning of the experimental session, participants filled out the consent forms and completed the pen test. The pen test is an unobtrusive, culture-sensitive test for assessing the preference for uniqueness vs. harmony adapted from Kim and Markus (1999, Study 3). We included the pen test as a manipulation check for culture (i.e., to ascertain that our participants were representative for their respective culture). Five ball pens were laid out on the table in an innocuous manner. Four pens had the same color (e.g., green) and one pen deviated in color (e.g., yellow). Participants chose one of the pens for filling out the questionnaires. Choosing the frequent color was interpreted as a preference for harmony, whereas choosing the infrequent color was interpreted as a preference for uniqueness (Kim and Markus, 1999, Study 3).

After the pen test, participants completed the Corsi block task (Corsi, 1972) in a computerized version (Rowe et al., 2009). The Corsi block task measures visuo-spatial working memory and was included as a measure of fluid cognition.^1^ Nine gray blocks are shown on a white background. In each trial, four to seven blocks turn black in a given sequence. The participants’ task is to reproduce the sequence by clicking on the blocks in the sequence they turned black. There were a total of 18 trials including 6 practice trials.

Then, the preparation for the EEG started. During this period, participants filled out a participant questionnaire containing medical and culture-related questions, the Edinburgh Handedness Inventory (Oldfield, 1971), and the Self-Construal-Scale (SCS; Singelis, 1994), which assesses independent and interdependent self-construal (Singelis, 1994; Singelis and Sharkey, 1995), in the updated version of Kitayama et al. (2014). We included the SCS as a manipulation check for culture (i.e., to ascertain that our participants were representative for their respective culture). After completing the EEG setup, the main experiment started, which will be described in detail in the next section.

Once participants completed the main experiment, a 10 min. Long pictorial classification task, in which participants had to indicate whether they see a line drawing of an object or a building (Mecklinger et al., 2014), followed. Since this task will be part of a different publication, it will not be further discussed in this manuscript.

After the classification task, participants completed the vocabulary test from the Wechsler Adult Intelligence Scale (WAIS; German version: Aster et al. (2006); Chinese version; Gong, 1983). This task was included to assess the crystalline component of cognition. At the end of the experiment, participants filled out a post-experimental questionnaire with several questions concerning task comprehension and compliance. Then participants were debriefed and received compensation for their participation.

### 2.3. Main experiment

#### 2.3.1. Stimulus material

**Figure 1:**
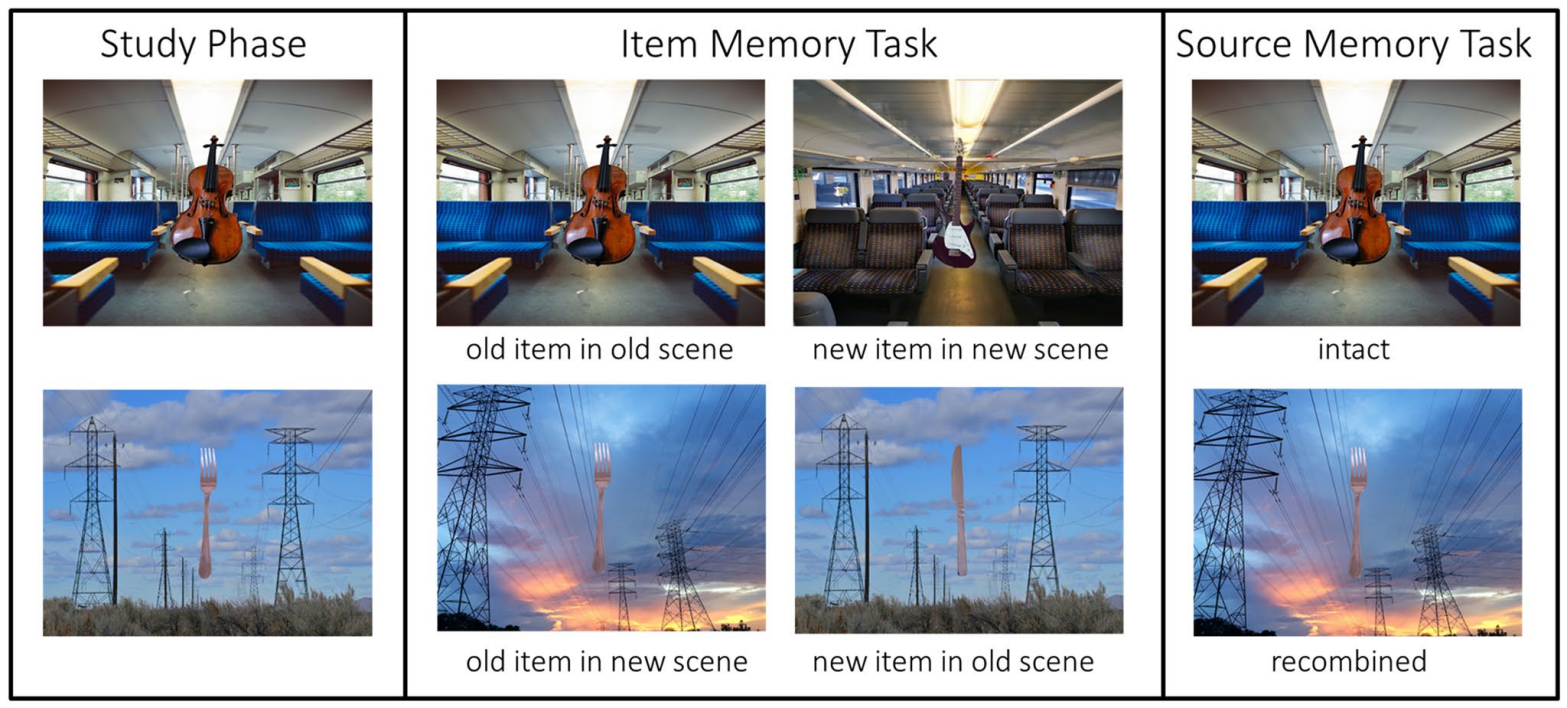
Example of the stimulus material.

The stimuli for the present study were taken from the ORCA (Official Rating of Complex Arrangements) database of object-scene arrangements (Weigl et al., 2023). Each selected quadruple contained four pictures (size: 640 × 480 pixels; see Figure 1, for an example of the material). Each picture featuring one of two highly familiar objects (e.g., two instruments) placed at the center of one of two scenes (e.g., two trains). Note that physical and semantic similarity was high for both instances of the object. The same was true for the scenes. Each object was placed in the center of the scene in order to reduce eye-movement artifacts (see Weigl et al., 2023 for more details on stimulus creation and stimulus properties).

**Figure 2:**
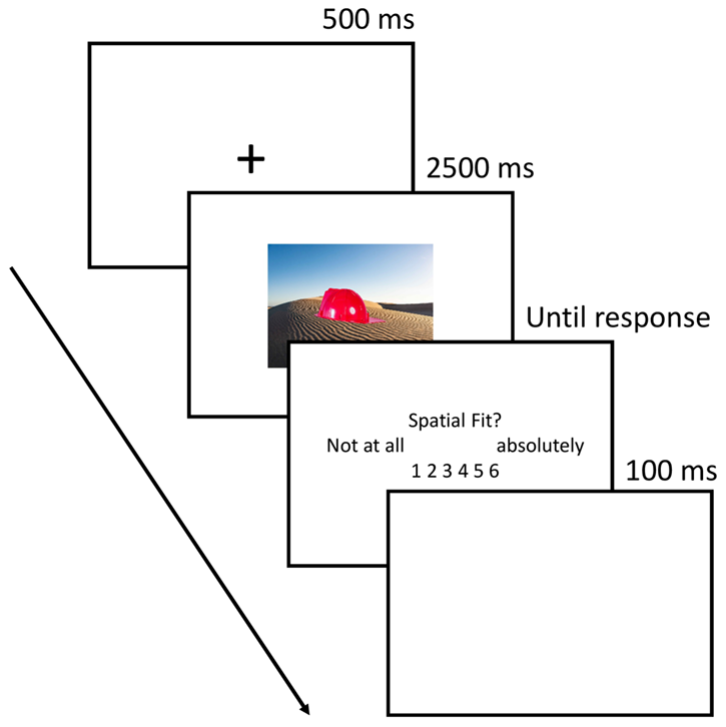
Procedure of the encoding task.

We selected 120 quadruples featuring semantically unrelated object-scene arrangements, which had a mean familiarity above 3.5 and a mean semantic fit below 3.5. The details on the stimulus material can be found in Table 2. Since the quadruples in the ORCA picture database were specifically created to provide intact and recombined object-scene pairs with high physical and semantic similarity (Weigl et al., 2023), intact and recombined pairs for this study were always drawn from the same quadruple. For example, a violin shown in front of a train with blue seats and an electric guitar shown in front of a train with gray seats would constitute the intact pairs (Figure 2). The recombined pairs showed the violin in front of the train with gray and the electric guitar in front of the train with blue seats.^2^ The rational for using recombined pairs was that recombining objects and backgrounds in the test phase allows us to directly test our main hypotheses, namely that Chinese participants should show worse item memory performance than German participants for old objects in new contexts, but both cultures should not differ in item memory performance for old items in the old context. For the source memory, we hypothesized that performance for old objects on the original background should be better for Chinese relative to German participants, whereas performance for old objects on a different background should be worse for Chinese relative to German participants.

**Table 2:** Information on the stimulus material selected from the ORCA picture database (Weigl et al., 2023).

	Germans	Chinese	Comparison
Familiarity	5.45 (0.32)	5.11 (0.41)	*t* (119) = 11.63, *p* < 0.001, Cohen’s d = 1.06
Semantic fit	1.79 (0.59)	1.59 (0.49)	*t* (119) = 4.87, *p* < 0.001, Cohen’s d = 0.44

Means (and standard deviations) for German and Chinese young adults are based on the data from the published norms.

#### 2.3.2. Procedure

The main experiment consisted of two item memory blocks and two source memory blocks. In order to prevent participants from using different encoding strategies during the main experiment, participants were told that the task order was randomly chosen by the computer prior to the main experiment. The participants received detailed instructions on the task via a PowerPoint presentation and completed two practice blocks– one for the item memory task and one for the source memory task. Each block in the practice phase contained eight items in the study phase and sixteen items in the test phase. The presentation and the practice blocks were used to familiarize participants with the task and to ensure that participants thought that the memory task was randomly selected.

The main experiment consisted of four study-test cycles, two subsequent blocks of item memory and two subsequent blocks of source memory. The order of item memory blocks and source memory blocks was counterbalanced across participants. Each cycle started with a rating task, which served as study phase, followed by a 2.5 min long filler oddball task.

##### 2.3.2.1. Study phase

Figure 2 depicts the procedure of the study phase. In the study phase, participants were instructed to rate each object for spatial fit with the background scene. Each study phase consisted of 30 trials. Each trial started with a 500 ms fixation cross. Then, the picture was presented for 2000 ms. After the picture offset, participants had to rate the spatial fit between the object and the background on a six-point scale (1 = not at all, 6 = absolutely). A self-paced approach was chosen in order to leave participants enough time for their judgment and to avoid missing values. After participants entered their response, a blank screen was shown for 100 ms and the next trial started.

##### 2.3.2.2. Oddball task

The oddball task was used as a filler task to provide a constant 2.5 min retention interval between the study and the test phase. Participants were presented a frequent standard stimulus (O) in 80% of the trials, a rare target stimulus (X) in 10% of the trials, and novel symbols (e.g., symbols not used in German or Chinese written language such as Ħ or Ѫ) in 10% of the trials. Each trial started with the presentation of the symbol for 200 ms, which was followed by a 1,300 ms fixation star. The task of the participants was to respond only to the target stimulus by pressing the space bar. The response time window was 1,500 ms starting with the onset of the symbol. The next trials started after the end of the response time window.

##### 2.3.2.3. Test phase

**Figure 3:**
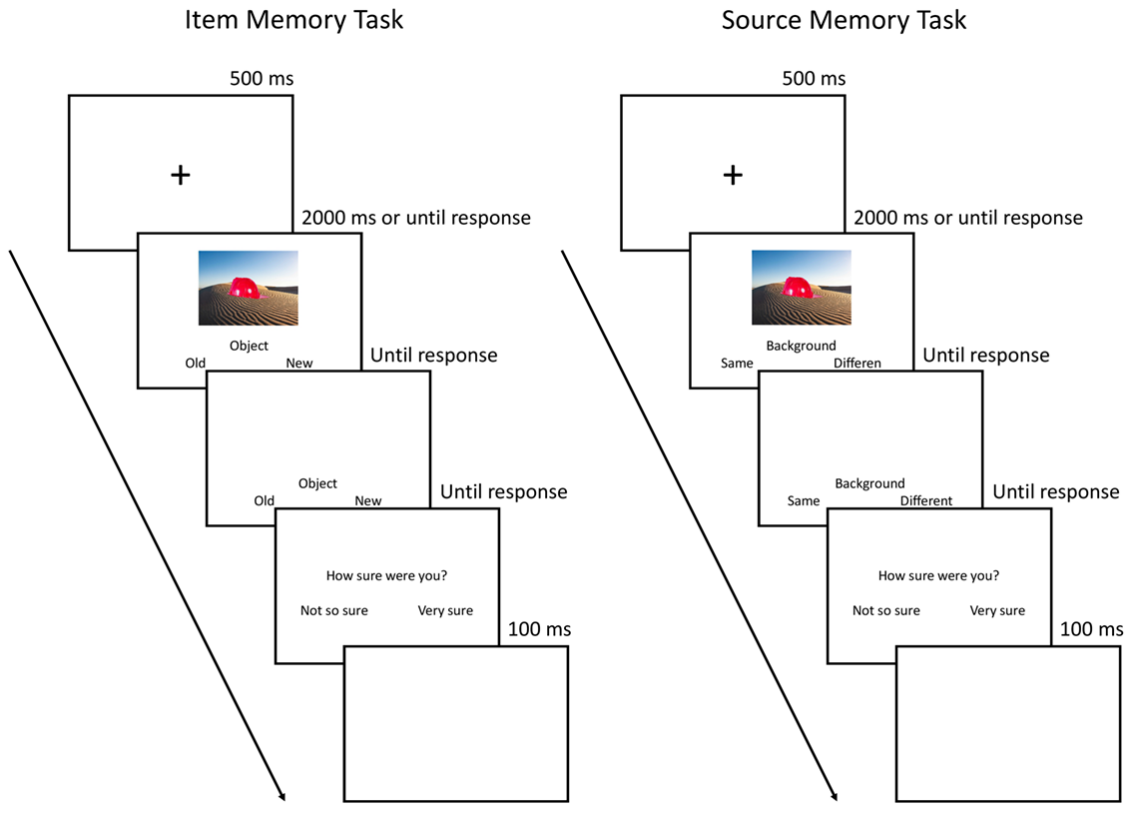
Procedure of the item and source memory blocks in the retrieval phase.

There were two types of memory tasks (see Figure 3): an item memory task, which tested memory for the focal object, and a source memory task, which tested for memory of the combination of object and scene. The trial structure of each type of memory task was as similar as possible. There were 60 trials irrespective of the memory condition. Each trial in the memory tasks started with a 500 ms fixation cross. Then, the picture was presented for 2000 ms.

In the item memory task, participants saw an old or new object on either an old or new background. After the picture appeared, the participants had to decide whether the object was old or new irrespective of the background scene by pressing F or J on a keyboard (with the index finger of the left and right hand, respectively). The response keys were counterbalanced across participants.

In the source memory task, participants only saw old objects placed on either the same scene as in the previous rating task or on a different scene. The different scene also was presented in the rating task, but paired with another object. After the picture appeared, the participants had to decide whether the object was paired with the same or a different background scene by pressing F or J on a keyboard (with the index finger of the left and right hand, respectively). The response keys were counterbalanced across participants.

There was no time limit for the response, even though the picture would disappear after 2000 ms if no response was made. Pilot data indicated that participants often needed more than 2,500 ms for their memory judgment – especially in the source memory task and for recombined pairs. As a strict time limit might have led to excessive missing values in certain conditions and such systematic missing values would hamper the validity of our data, we preferred a self-paced approach over a pre-determined time limit. After the memory decision, participants had to indicate their confidence, i.e., whether they were “not so sure” or “very sure” by pressing F or J, respectively (again using the index finger of their left and right hand, respectively). The next trial started after a 100 ms blank.

### 2.4. EEG

#### 2.4.1. EEG recoding

On the German side, EEG was recorded with an elastic cap (Easycap, Herrsching, Germany) with 28 embedded Ag/AgCl EEG electrodes (recording sites: Fp1, Fp2, F7, F3, Fz, F4, F8, FC5, FC3, FCz, FC4, FC6, T7, C3, Cz, C4, T8, CP3, CPz, CP4, P7, P3, Pz, P4, P8, O1, O2, and the right mastoid M2; ground: AFz; reference: left mastoid M1). EOG activity was recorded with two electrodes placed on the outer canthi and by a pair of electrodes placed above and below the right eye. BrainVision Recorder 1.0 (BrainProducts, Gilching, Germany) was used for data recording. Data were sampled at 500 Hz and filtered online from 0.016 Hz to 250 Hz. Electrode impedance was kept below 5kΩ for the whole session.

On the Chinese side, EEG was recorded with an elastic cap (*Neuroscan*, *Charlotte, North Carolina, USA*) with 63 embedded Ag/AgCl EEG electrodes (recording sites: Fp1, Fpz, Fp2, AF3, AF4, F7, F5, F3, F1, Fz, F2, F4, F6, F8, FT7, FC5, FC3, FC1, FCz, FC2, FC4, FC6, FT8, T7, C5, C3, C1, Cz, C2, C4, C6, T8, TP7, CP5, CP3, CP1, CPz, CP2, CP4, CP6, TP8, P7, P5, P3, P1, Pz, P2, P4, P6, P8, PO7, PO5, PO3, POz, PO4, PO6, PO8, CB1, O1, Oz, O2, CB2, and the right mastoid M2; ground: AFz; reference: left mastoid M1). EOG activity was recorded with two electrodes placed on the outer canthi and by a pair of electrodes placed above and below the right eye. NeuroScan Acquire 4.3.1 software was used for data recording. Data were sampled at 500 Hz and filtered online from 0.05 to 100 Hz. Electrode impedance was kept below 5kΩ for the whole session.

#### 2.4.2. ERP data processing

EEG data from both cultures were processed in Brain Vision Analyzer 2.1 (Brain Products, Gilching, Germany) with the same preprocessing protocol to warrant maximal comparability of the ERP results. In order to match the number of electrodes during preprocessing, electrode positions for the Chinese data, which were not recorded on the German side, were removed from further processing. Data preprocessing started with a 0.05 Hz high pass filter (order 8). Then, an independent component analysis (ICA) was applied to remove ocular, electrocardiographic (ECG), and muscle artifacts. Next, data were filtered with a 30 Hz low-pass filter (order 8) and re-referenced to linked mastoids. After segmenting the data into 2,200 ms epochs (including a 200 ms baseline period) and baseline correction, segments with amplitudes exceeding ±100 μV were removed from analysis. Then, data were averaged separately for each condition. Finally, mean amplitudes were calculated for the time windows 300–500 ms, 500–800 ms, and 800–1,200 ms.

### 2.5. Data analysis

Participants with less than 15 trials in one of the conditions of the memory blocks or with less than 80% of the trials (indicating overall poor data quality) were excluded from all analyzes. All mixed-design (M) ANOVAs were conducted with SPSS 28. All Bayesian t-tests were conducted with JASP 0.17.1 (Love et al., 2019).

#### 2.5.1. Behavioral

For both, the item and the source memory task, we analyzed the accuracies (ACCs; i.e. hits and correct rejections) and the discrimination index Pr (= hits–false alarms).^3^ The ACCs and the RTs for were analyzed with separate mixed-design ANOVAs with Culture (German vs. Chinese) as a between-subject factor, Object (Old vs. New) and Background (Old vs. New) as within-subject factors. In order to assess cultural differences in discrimination and response bias in item memory, separate mixed-design ANOVAs with Culture (German vs. Chinese) as a between-subject factor and Background (Old vs. New) as within-subject factors were calculated for the discrimination index Pr (Snodgrass and Corwin, 1988).

The ACCs for the source memory task were analyzed with separate mixed-design ANOVA with Culture (German vs. Chinese) as a between-subject factor and Background (Old vs. New) as within-subject factors. In order to assess cultural differences in discrimination in source memory, separate ANOVAs with Culture (German vs. Chinese) as a between-subject factor were calculated for Pr.

#### 2.5.2. ERPs

We chose MANOVA (multivariate ANOVA) for the analysis of the ERP data, because this procedure does not assume sphericity and is therefore more powerful for ERP data than traditional ANOVA (Handy, 2005). Time windows were determined based on the literature (e.g., Rugg and Curran, 2007; Mecklinger et al., 2016). All analyzes used the electrodes F3, Fz, F4, C3, Cz, C4, P3, Pz, and P4.

For the item memory task, the mean amplitudes for the 300–500 ms time window, the 500–800 ms time window, and the 800–1,200 ms time window were subjected to separate MANOVAs with Culture (German vs. Chinese) as a between-subject factor and AnteriorPosterior (Frontal vs. Central vs. Parietal), Laterality (Left vs. Middle vs. Right), Item (Old vs. New), and Background (Old vs. New) as within-subject factors.

For the source memory task, the mean amplitudes for the 300–500 ms time window, the 500–800 ms time window, and the 800–1,200 ms time window were subjected to separate MANOVAs with Culture as a between-subject factor and AnteriorPosterior (Frontal vs. Central vs. Parietal), Laterality (Left vs. Middle vs. Right), and Source (Intact vs. Recombined) as within-subject factors.

Please note that mere topographical main effects or interactions between the AnteriorPosterior and Laterality factors will not be discussed in the result section as these are not related to our main hypotheses.

#### 2.5.3. Bayesian *t*-tests

In order to test, whether potential null results stem from inconclusive data or actual evidence for the null hypothesis, we also conducted Bayesian t-tests as a complementary statistical approach to our data. Conventional (frequentist) t-tests produce a value of p, which expresses the probability of the data given the null hypothesis. Thus, frequentist tests only allow researchers to accept or reject the null hypothesis. By contrast, the Bayesian t-test allows researchers to address the more relevant question whether their data provides evidence for the null hypothesis or the alternative hypothesis, or whether their data are inconclusive by looking at the Bayes factor (Rouder et al., 2009). This approach compensates for one weakness of frequentist null-hypothesis testing, namely the limited usefulness of the value of p.

The Bayes factor BF, a metric to evaluate evidence for or against a specific hypothesis, was used for interpretation. A BF_10_ > 1 (or BF_01_ < 1) means that there is more evidence in favor for the alternative hypothesis than for the null hypothesis, whereas a BF_10_ < 1 (or BF_01_ > 1) means that there is more evidence in favor for the null hypothesis than for the alternative hypothesis (van den Bergh et al., 2020). Moreover, BF_10_ = BF_01_ = 1 means that there is as much evidence for the null hypothesis as there is evidence for the alternative hypothesis. In other words, the Bayes factor allows us to distinguish whether our data are nondiagnostic or favor a particular hypothesis (null or alternative hypothesis)^4^.

Default priors were used for all analyzes. We formally interpreted BF_10_ > 3 as substantial evidence for H_1_ and BF_10_ < 1/3 as substantial evidence for H_0_ (Jeffreys, 1961)^5^. For the behavioral data, the differences in accuracy and the Pr scores were subjected to an Bayesian independent sample t-test with Culture as grouping variable. For the ERP data, old/new effects for each of the nine electrodes (F3, Fz, F4, C3, Cz, C4, P3, Pz, and P4), time window (300–500 ms, 500–800 ms, and 800–1,200 ms), and tasks (old background, new background, and source) were subjected to a Bayesian independent sample t-test with Culture as grouping variable.

## 3. Results

### 3.1. Sample characteristics

The results for the demographic, cultural, and neuropsychological data can be found in Table 3. While no significant differences were observed for age and gender ratio, Chinese participants had more years of education than German participants. Moreover, we found cultural differences in the pen test and the SCS. As expected, German participants preferred the rare pen, whereas the reverse was true for Chinese participants. Self-construal was more independent in Germans than in Chinese. No difference was observed in interdependent self-construal. Overall, these results suggest that our sample was comparable in terms of demographic and exhibited cultural differences in the expected direction in the cultural variables.

**Table 3:** Demographic information on our sample.

	Germans (*N* = 25)	Chinese (*N* = 32)	Comparison
Mean age (SD)	21.4 y/o (1.5 y/o)	22.2 y/o (2.6 y/o)	*t* (51.28) = −1.31, *p* = 0.195, Cohen’s d = −0.33
Gender (M/F)	7/18	6/26	*χ*^2^ (1) = 0.65, *p* = 0.409, φ = 0.11
Years of education	14.8 yrs. (1.5 yrs)	16.3 yrs. (2.6 yrs)	*t* (51.10) = −2.74, *p* = 0.009, Cohen’s d = −0.69
SCS Independence	4.37 (0.57)	3.95 (0.50)	*t* (55) = 2.94, *p* = 0.005, Cohen’s d = 0.79
SCS Interdependence	3.73 (0.74)	3.87 (0.49)	*t* (39.56) = −0.79, *p* = 0.410, Cohen’s d = −0.22
Pen Test (rare/common)	15/10	9/23	*χ*^2^ (1) = 5.85, *p* = 0.016, φ = 0.32

For continuous variables, mean and standard deviations are reported as descriptive statistics and t-test for comparison. For categorical variable, frequencies are reported as descriptive statistics and *χ*^2^ test for comparison.

### 3.2. Behavioral results

#### 3.2.1. Item memory

The descriptive statistics for the item memory task can be found in Table 4. The mixed-design ANOVA for the accuracy data (Hits and CRs) revealed significant main effects for Object (*F* (1, 55) = 29.46, *p* < 0.001, *η*_p_^2^ = 0.35) and for Background (F (1, 55) = 15.15, p < 0.001, *η*_p_^2^ = 0.22), a marginally significant main effect for Culture (F (1, 55) = 3.80, *p* = 0.056, *η*_p_^2^ = 0.07), and interactions between Object and Culture (F (1, 55) = 7.01, *p* = 0.011, *η*_p_^2^ = 0.11) and between Object and Background (F (1, 55) = 41.94, p < 0.001, *η*_p_^2^ = 0.43). The interactions between Background and Culture and between Object, Background, and Culture were not significant (F (1, 55) = 0.00, *p* = 0.959, *η*_p_^2^ = 0.00 and F (1, 55) = 0.02, *p* = 0.903, *η*_p_^2^ = 0.00, respectively).

**Table 4:** Mean and standard deviation for the accuracies and Pr scores in the item and source memory task.

Task	Measure	Type	Germans	Chinese
Item memory	Accuracy	Old item old background	0.83 (0.15)	0.90 (0.07)
		Old item new background	0.74 (0.13)	0.81 (0.11)
		New item old background	0.88 (0.10)	0.88 (0.08)
		New item new background	0.90 (0.07)	0.90 (0.07)
	Pr	Old background	0.71 (0.20)	0.78 (0.11)
		New background	0.64 (0.17)	0.71 (0.15)
Source memory	Accuracy	Intact	0.73 (0.10)	0.75 (0.07)
		Recombined	0.59 (0.11)	0.55 (0.12)
	Pr	–	0.32 (0.13)	0.30 (0.16)

The follow-up ANOVA for hits revealed a main effect for Background (F (1, 55) = 43.60, p < 0.001, *η*_p_^2^ = 0.44), and for Culture (F (1, 55) = 6.78, *p* < 0.012, *η*_p_^2^ = 0.11), indicating that hits were higher for old backgrounds relative to new backgrounds and for Chinese participants relative to German participants. The interaction between Background and Culture was not significant (F (1, 55) = 0.01, *p* = 0.912, *η*_p_^2^ = 0.00).

The follow-up ANOVA for CRs revealed a trend for Background (F (1, 55) = 2.87, *p* = 0.096, *η*_p_^2^ = 0.43), suggesting that CRs tended to be higher for new objects presented on new backgrounds relative to new objects presented on old backgrounds. Neither the main effect for Culture (F (1, 55) = 0.00, *p* = 0.961, *η*_p_^2^ = 0.00) nor the interaction between Background and Culture (F (1, 55) = 0.04, *p* = 0.844, *η*_p_^2^ = 0.00) were significant.

The analysis of the Pr scores revealed a main effect for background (F (1, 55) = 15.15, p < 0.001, *η*_p_^2^ = 0.22) and a marginally significant main effect for Culture (F (1, 55) = 3.80, p = 0.056, *η*_p_^2^ = 0.07), showing that object discrimination was better for old backgrounds relative to new backgrounds and that Chinese participants tended to perform better than German participants. The predicted interaction between Background and Culture, however, was not significant (F (1, 55) = 0.00, p = 0.959, *η*_p_^2^ = 0.00).

#### 3.2.2. Source memory

The descriptive statistics for the source memory task can be found in Table 4. The analysis of the accuracy data revealed a main effect for Background (F (1, 55) = 74.04, p < 0.001, *η*_p_^2^ = 0.57), indicating that accuracy was higher for intact than for recombined stimuli. Neither the main effect for Culture (F (1, 55) = 0.14, *p* = 0.709, *η*_p_^2^ = 0.00) nor the interaction between Background and Culture (F (1, 55) = 1.98, *p* = 0.165, *η*_p_^2^ = 0.04) were significant. Also, the analysis of the Pr scores did not reveal any difference between intact and recombined stimuli (F (1, 55) = 0.14, *p* = 0.709, *η*_p_^2^ = 0.00).

#### 3.2.3. Bayesian analyzes

In order to explore whether the non-significant results actually reflect evidence for the null hypotheses, Bayesian t-tests were conducted for the predicted effects. The BF values for the hypothesis-relevant comparisons in the item and source memory tasks can be found in Table 5. As indicated by the BF, there is substantial evidence for the H_0_ for all comparisons except for the comparison between intact and recombined stimuli in the source memory task, which provided only anectodical evidence for the H_0_.

**Table 5:** Bayes factors for *t*-test with culture as independent variable and the critical item and source memory comparisons as dependent variable.

Comparison	BF_10_	BF_01_	Interpretation
Hit_OldBackground – Hit_NewBackground	0.27	3.69	Substantial evidence for H_0_
CR_OldBackground – CR_NewBackground	0.27	3.70	Substantial evidence for H_0_
Pr_OldBackground – Pr_NewBackground	0.27	3.70	Substantial evidence for H_0_
Intact–Recombined	0.61	1.63	Anecdotal evidence for H_0_
Pr_Source	0.29	3.50	Substantial evidence for H_0_

#### 3.2.4. Summary

To sum up, item memory was better than source memory in both cultures. However, the behavioral data gave no indication that item memory or source memory performance for contexts was modulated by culture. In fact, the Bayesian analyzes indicated that our data provide evidence in favor for the null hypothesis.

### 3.3. ERP results

#### 3.3.1. ERPs for the item memory task

**Figure 4:**
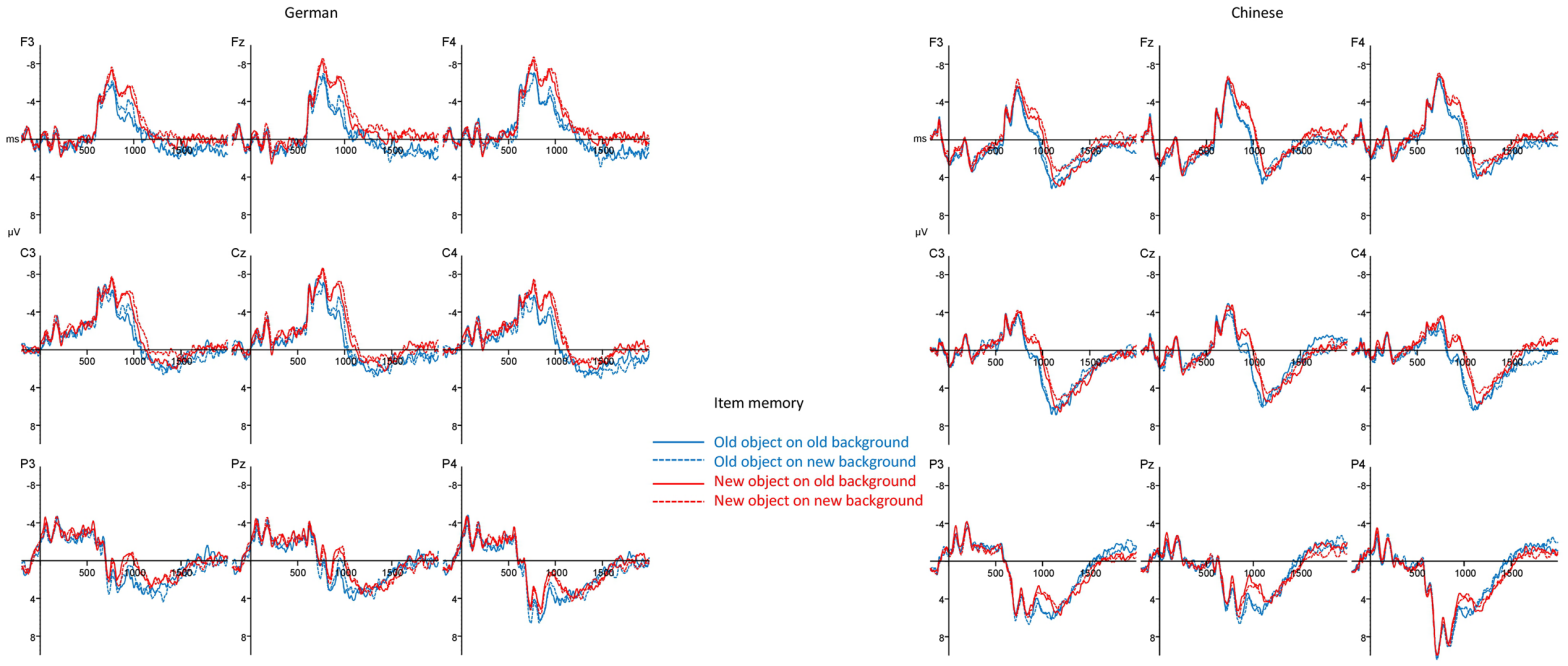
ERPs for the item memory blocks (negativity plotted upwards).

The ERP waveforms for the item memory task for both cultures can be seen in Figure 4. In general, the waveforms of the Chinese participants are more positive than the waveforms of the German participants. Overall, an old/new effect starting in the 500–800 ms time window can be seen in both cultures. This effect persists through the 800–1,200 ms time window. In this time window, some effects for the background emerge. However, culture did not modulate any of the observed effects. This visual impression was corroborated by the statistical analyzes. Please refer to Table 6 for an overview over the results for the item memory task in each of the three time windows.

**Table 6:** Results for the MANOVA in the item memory block.

	300–500 ms	500–800 ms	800–1,200 ms
AnteriorPosterior	F (2,54) = 17.934 *p* < 0.001, *η*_p_^2^ = 0.399	*F* (2,54) = 85.586 *p* < 0.001, *η*_p_^2^ = 0.760	F (2,54) = 54.197 *p* < 0.001, *η*_p_^2^ = 0.667
AnteriorPosterior*Culture	F (2,54) = 4.248 *p* = 0.019, *η*_p_^2^ = 0.136	F (2,54) = 5.248 *p* = 0.008, *η*_p_^2^ = 0.163	F (2,54) = 9.685 p < 0.001, *η*_p_^2^ = 0.264
Laterality	F (2,54) = 27.733 *p* < 0.001, *η*_p_^2^ = 0.507	F (2,54) = 11.139 *p* < 0.001, *η*_p_^2^ = 0.292	F (2,54) = 4.953 *p* = 0.011, *η*_p_^2^ = 0.155
Laterality*Culture	F (2,54) = 3.854 *p* = 0.027, *η*_p_^2^ = 0.125	F (2,54) = 0.360 *p* = 0.700, *η*_p_^2^ = 0.013	F (2,54) = 1.163 *p* = 0.320, *η*_p_^2^ = 0.041
Item	F (1,55) = 0.491 *p* = 0.486, *η*_p_^2^ = 0.009	F (1,55) = 2.716 *p* = 0.105, *η*_p_^2^ = 0.047	F (1,55) = 23.644 *p* < 0.001, *η*_p_^2^ = 0.301
Item*Culture	F (1,55) = 0.320 *p* = 0.574, *η*_p_^2^ = 0.006	F (1,55) = 0.148 *p* = 0.702, *η*_p_^2^ = 0.003	F (1,55) = 0.123 *p* = 0.727, *η*_p_^2^ = 0.002
Background	F (1,55) = 0.026 *p* = 0.872, *η*_p_^2^ = 0.000	F (1,55) = 0.260 *p* = 0.612, *η*_p_^2^ = 0.005	F (1,55) = 1.266 *p* = 0.265, *η*_p_^2^ = 0.022
Background*Culture	F (1,55) = 0.030 *p* = 0.864, *η*_p_^2^ = 0.001	F (1,55) = 0.394 *p* = 0.533, *η*_p_^2^ = 0.744	F (1,55) = 0.105 *p* = 0.747, *η*_p_^2^ = 0.002
AnteriorPosterior*Laterality	F (4,52) = 14.206 *p* < 0.001, *η*_p_^2^ = 0.522	F (4,52) = 37.867 *p* < 0.001, *η*_p_^2^ = 0.744	F (4,52) = 18.786 *p* < 0.001, *η*_p_^2^ = 0.591
AnteriorPosterior*Laterality*Culture	F (4,52) = 1.798 *p* = 0.143, *η*_p_^2^ = 0.122	F (4,52) = 0.588 *p* = 0.672, *η*_p_^2^ = 0.043	F (4,52) = 1.017 *p* = 0.407, *η*_p_^2^ = 0.073
AnteriorPosterior*Item	F (2,54) = 0.104 *p* = 0.901, *η*_p_^2^ = 0.004	F (2,54) = 0.661 *p* = 0.521, *η*_p_^2^ = 0.024	F (2,54) = 5.548 *p* = 0.006, *η*_p_^2^ = 0.170
AnteriorPosterior*Item*Culture	F (2,54) = 1.372 *p* = 0.262, *η*_p_^2^ = 0.048	F (2,54) = 0.821 *p* = 0.445, *η*_p_^2^ = 0.030	F (2,54) = 0.850 *p* = 0.433, *η*_p_^2^ = 0.031
Laterality*Item	F (2,54) = 0.557 *p* = 0.576, *η*_p_^2^ = 0.020	F (2,54) = 0.513 *p* = 0.601, *η*_p_^2^ = 0.019	F (2,54) = 1.817 *p* = 0.172, *η*_p_^2^ = 0.063
Laterality*Item*Culture	F (2,54) = 0.420 *p* = 0.659, *η*_p_^2^ = 0.015	F (2,54) = 0.047 *p* = 0.955, *η*_p_^2^ = 0.002	F (2,54) = 0.704 *p* = 0.499, *η*_p_^2^ = 0.025
AnteriorPosterior*Laterality*Item	F (4,52) = 1.675 *p* = 0.170, *η*_p_^2^ = 0.114	F (4,52) = 2.623 *p* = 0.045, *η*_p_^2^ = 0.168	F (4,52) = 2.364 *p* = 0.065, *η*_p_^2^ = 0.154
AnteriorPosterior*Laterality*Item*Culture	F (4,52) = 2.530 *p* = 0.051, *η*_p_^2^ = 0.163	F (4,52) = 1.610 *p* = 0.186, *η*_p_^2^ = 0.110	F (4,52) = 1.537 *p* = 0.205, *η*_p_^2^ = 0.106
AnteriorPosterior*Background	F (2,54) = 0.063 *p* = 0.939, *η*_p_^2^ = 0.002	F (2,54) = 0.128 *p* = 0.880, *η*_p_^2^ = 0.005	F (2,54) = 3.721 *p* = 0.031, *η*_p_^2^ = 0.121
AnteriorPosterior*Background*Culture	F (2,54) = 0.489 *p* = 0.616, *η*_p_^2^ = 0.018	F (2,54) = 0.083 *p* = 0.920, *η*_p_^2^ = 0.003	F (2,54) = 1.043 *p* = 0.359, *η*_p_^2^ = 0.037
Laterality*Background	F (2,54) = 0.232 *p* = 0.794, *η*_p_^2^ = 0.009	F (2,54) = 0.757 *p* = 0.474, *η*_p_^2^ = 0.027	F (2,54) = 1.133 *p* = 0.330, *η*_p_^2^ = 0.040
Laterality*Background*Culture	F (2,54) = 0.895 *p* = 0.414, *η*_p_^2^ = 0.032	F (2,54) = 0.351 *p* = 0.706, *η*_p_^2^ = 0.013	F (2,54) = 2.805 *p* = 0.069, *η*_p_^2^ = 0.094
AnteriorPosterior*Laterality*Background	F (4,52) = 1.579 *p* = 0.194, *η*_p_^2^ = 0.108	F (4,52) = 1.176 *p* = 0.332, *η*_p_^2^ = 0.083	F (4,52) = 0.382 *p* = 0.820, *η*_p_^2^ = 0.029
AnteriorPosterior*Laterality*Background*Culture	F (4,52) = 0.166 *p* = 0.955, *η*_p_^2^ = 0.013	F (4,52) = 0.243 *p* = 0.912, *η*_p_^2^ = 0.018	F (4,52) = 0.008 *p* = 1.000, *η*_p_^2^ = 0.001
Item*Background	F (1,55) = 0.348 *p* = 0.558, *η*_p_^2^ = 0.006	F (1,55) = 0.508 *p* = 0.479, *η*_p_^2^ = 0.009	F (1,55) = 0.049 *p* = 0.826, *η*_p_^2^ = 0.001
Item*Background*Culture	F (1,55) = 0.064 *p* = 0.801, *η*_p_^2^ = 0.001	F (1,55) = 0.455 *p* = 0.503, *η*_p_^2^ = 0.008	F (1,55) = 0.112 *p* = 0.739, *η*_p_^2^ = 0.002
AnteriorPosterior*Item*Background	F (2,54) = 0.408 *p* = 0.667, *η*_p_^2^ = 0.015	F (2,54) = 2.368 *p* = 0.103, *η*_p_^2^ = 0.081	F (2,54) = 2.350 p = 0.105, *η*_p_^2^ = 0.080
AnteriorPosterior*Item*Background*Culture	F (2,54) = 0.140 *p* = 0.869, *η*_p_^2^ = 0.005	F (2,54) = 0.299 *p* = 0.743, *η*_p_^2^ = 0.011	F (2,54) = 0.523 *p* = 0.596, *η*_p_^2^ = 0.080
Laterality*Item*Background	F (2,54) = 1.604 *p* = 0.210, *η*_p_^2^ = 0.056	F (2,54) = 1.413 *p* = 0.252, *η*_p_^2^ = 0.050	F (2,54) = 0.402 *p* = 0.671, *η*_p_^2^ = 0.015
Laterality*Item*Background*Culture	F (2,54) = 0.363 *p* = 0.697, *η*_p_^2^ = 0.013	F (2,54) = 0.165 *p* = 0.848, *η*_p_^2^ = 0.006	F (2,54) = 0.664 *p* = 0.519, *η*_p_^2^ = 0.024
AnteriorPosterior*Laterality*Item*Background	F (4,52) = 0.246 *p* = 0.911, *η*_p_^2^ = 0.019	F (4,52) = 0.729 p = 0.576, *η*_p_^2^ = 0.053	F (4,52) = 0.737 *p* = 0.571, *η*_p_^2^ = 0.054
AnteriorPosterior*Laterality*Item*Background*Culture	F (4,52) = 1.998 *p* = 0.109, *η*_p_^2^ = 0.133	F (4,52) = 2.036 *p* = 0.103, *η*_p_^2^ = 0.135	F (4,52) = 2.154 *p* = 0.087, *η*_p_^2^ = 0.142
Culture	F (1,55) = 4.349 *p* = 0.042, *η*_p_^2^ = 0.073	F (1,55) = 5.251 *p* = 0.026, *η*_p_^2^ = 0.087	F (1,55) = 6.140 *p* = 0.016, *η*_p_^2^ = 0.100

Significant main effects and interactions are marked with gray.

In the 300–500 ms time window, Culture was the only non-topographical factor, which became significant as a main effect and in interaction with the topographical factors AnteriorPosterior and Laterality. In other words, ERPs from Chinese participants were more positive than ERPs from German participants. No old/new effect was observed in this early time window.

In the 500–800 ms time window, ERPs from the Chinese participants were again more positive than ERPs from the German participants. There was also a significant interaction between AnteriorPosterior, Laterality, and Item, indicating that the ERPs were more positive for old than new foreground objects. Contrary to our expectations and the literature, this old/new effect had a more fronto-central distribution than the late parietal old/new effect typically observed in this time window. Moreover, the old/new effect for the focal objects was not modulated by culture.

In the 800–1,200 ms time window, ERPs from Chinese participants were again more positive than ERPs from German participants and the old/new effect for the focal item remained significant, too (i.e., the AnteriorPosterior x Item interaction). In addition, there was a significant interaction between AnteriorPosterior, Laterality, and Background. The waveforms were more positive for old backgrounds than for new backgrounds. This effect was most pronounced at left fronto-central electrodes and indicates the presence of an old/new effect for the background scene in this late time interval. None of the old/new effect was modulated by culture.

#### 3.3.2. ERPs for the source memory task

**Figure 5:**
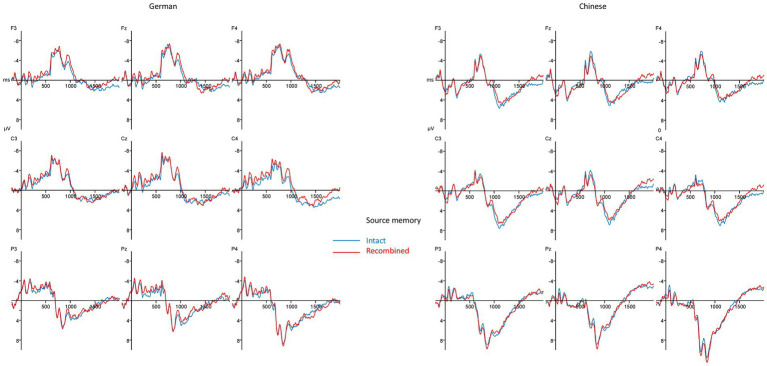
ERPs for the source memory blocks (negativity plotted upwards).

The ERP waveforms for the source memory task of both culture groups can be seen in Figure 5. In general, the waveforms of the Chinese participants are more positive than the waveforms of the German participants. Contrary to our predictions, no particularly strong differences between intact and recombined items are visible in the waveforms of this task. This visual impression was corroborated by the statistical analyzes. Please refer to Table 7 for an overview over the results for the source memory task.

**Table 7:** Results for the MANOVA in source memory block.

	300–500 ms	500–800 ms	800–1,200 ms
AnteriorPosterior	F (2,54) = 17.970 *p* < 0.001, *η*_p_^2^ = 0.400	F (2,54) = 109.174 *p* < 0.001, *η*_p_^2^ = 0.802	F (2,54) = 53.697 *p* < 0.001, *η*_p_^2^ = 0.665
AnteriorPosterior*Culture	F (2,54) = 3.127 *p* = 0.052, *η*_p_^2^ = 0.104	F (2,54) = 2.982 *p* = 0.059, *η*_p_^2^ = 0.099	F (2,54) = 12.287 p < 0.001, *η*_p_^2^ = 0.313
Laterality	F (2,54) = 27.750 *p* < 0.001, *η*_p_^2^ = 0.507	F (2,54) = 19.239 *p* < 0.001, *η*_p_^2^ = 0.416	F (2,54) = 1.906 *p* = 0.158, *η*_p_^2^ = 0.966
Laterality*Culture	F (2,54) = 6.213 *p* = 0.004, *η*_p_^2^ = 0.187	F (2,54) = 0.478 *p* = 0.623, *η*_p_^2^ = 0.017	F (2,54) = 3.162 *p* = 0.050, *η*_p_^2^ = 0.105
Source	F (1,55) = 0.353 *p* = 0.555, *η*_p_^2^ = 0.006	F (1,55) = 0.000 *p* = 0.987, *η*_p_^2^ = 0.000	F (1,55) = 2.711 p = 0.105, *η*_p_^2^ = 0.047
Source*Culture	F (1,55) = 4.054 *p* = 0.049, *η*_p_^2^ = 0.069	F (1,55) = 3.137 *p* = 0.082, *η*_p_^2^ = 0.054	F (1,55) = 0.078 *p* = 0.781, *η*_p_^2^ = 0.001
AnteriorPosterior*Laterality	F (4,52) = 6.980 *p* < 0.001, *η*_p_^2^ = 0.349	F (4,52) = 30.030 *p* < 0.001, *η*_p_^2^ = 0.698	F (4,52) = 17.335 *p* < 0.001, *η*_p_^2^ = 0.571
AnteriorPosterior*Laterality*Culture	F (4,52) = 0.704 *p* = 0.593, *η*_p_^2^ = 0.051	F (4,52) = 0.201 *p* = 0.937, *η*_p_^2^ = 0.015	F (4,52) = 1.735 *p* = 0.156, *η*_p_^2^ = 0.118
AnteriorPosterior*Source	F (2,54) = 0.622 *p* = 0.541, *η*_p_^2^ = 0.023	F (2,54) = 0.671 *p* = 0.515, *η*_p_^2^ = 0.024	F (2,54) = 1.831 p = 0.170, *η*_p_^2^ = 0.064
AnteriorPosterior*Source*Culture	F (2,54) = 1.288 *p* = 0.284, *η*_p_^2^ = 0.046	F (2,54) = 0.361 *p* = 0.699, *η*_p_^2^ = 0.013	F (2,54) = 0.152 *p* = 0.859, *η*_p_^2^ = 0.006
Laterality*Source	F (2,54) = 0.184 *p* = 0.833, *η*_p_^2^ = 0.046	F (2,54) = 0.364 *p* = 0.697, *η*_p_^2^ = 0.013	F (2,54) = 0.129 *p* = 0.879, *η*_p_^2^ = 0.005
Laterality*Source*Culture	F (2,54) = 1.096 *p* = 0.342, *η*_p_^2^ = 0.039	F (2,54) = 1.006 *p* = 0.372, *η*_p_^2^ = 0.036	F (2,54) = 0.928 *p* = 0.401, *η*_p_^2^ = 0.033
AnteriorPosterior*Laterality*Source	F (4,52) = 2.323 *p* = 0.069, *η*_p_^2^ = 0.152	F (4,52) = 1.610 *p* = 0.186, *η*_p_^2^ = 0.110	F (4,52) = 1.791 *p* = 0.145, *η*_p_^2^ = 0.121
AnteriorPosterior*Laterality*Source*Culture	F (4,52) = 1.773 *p* = 0.148, *η*_p_^2^ = 0.120	F (4,52) = 1.059 *p* = 0.386, *η*_p_^2^ = 0.075	F (4,52) = 1.460 *p* = 0.228, *η*_p_^2^ = 0.101
Culture	F (1,55) = 9.289 *p* = 0.004, *η*_p_^2^ = 0.144	F (1,55) = 8.542 *p* = 0.005, *η*_p_^2^ = 0.134	F (1,55) = 7.775 *p* = 0.007, *η*_p_^2^ = 0.124

Significant main effects and interactions are marked with gray.

In the 300–500 ms time window, there was a significant interaction between Source and Culture. This interaction reflects the fact that the waveforms were more positive going for intact than for recombined object-background combinations in German participants, but not in Chinese participants. However, this early effect was widespread across the scalp, and therefore differs topographically from the early mid-frontal old/new effect.

Neither the analysis for the 500–800 ms time window nor for the 800–1,200 ms time window revealed any significant main effects or interactions involving the Source factor, i.e., no old/new effects were observed in the middle and late time windows. Only the Culture factor was significant alone and/or in interaction with the topographical factors, indicating that the ERPs from Chinese participants were more positive than ERPs from German participants.

#### 3.3.3. Bayesian analyzes

The results for the Bayesian analyzes of the old/new effects in the item memory task can be found in Table 8 for the items presented in front of the old background and in Table 9 for the items presented in front of the new background. Consistent with the visual impression and the frequentist analyzes, the Bayesian *t*-tests revealed that there is substantial evidence for the H_0_ for the majority of electrodes across all three time windows (15 out of 54, 72%) and at least anecdotal evidence for the H_0_ for the remaining electrodes.

**Table 8:** Bayes factors for critical comparisons in the ERP old/new effects for old backgrounds in the item memory blocks.

Time window	Electrode	BF_10_	BF_01_	Interpretation
300–500	F3	0.274	3.656	Substantial evidence for H_0_
	Fz	0.277	3.611	Substantial evidence for H_0_
	F4	0.321	3.111	Substantial evidence for H_0_
	C3	0.270	3.706	Substantial evidence for H_0_
	Cz	0.355	2.819	Substantial evidence for H_0_
	C4	0.271	3.684	Substantial evidence for H_0_
	P3	0.339	2.950	Anecdotal evidence for H_0_
	Pz	0.302	3.309	Substantial evidence for H_0_
	P4	0.357	2.803	Anecdotal evidence for H_0_
500–800	F3	0.282	3.541	Substantial evidence for H_0_
	Fz	0.317	3.150	Substantial evidence for H_0_
	F4	0.330	3.032	Substantial evidence for H_0_
	C3	0.336	2.975	Anecdotal evidence for H_0_
	Cz	0.274	3.650	Substantial evidence for H_0_
	C4	0.300	3.332	Substantial evidence for H_0_
	P3	0.281	3.555	Substantial evidence for H_0_
	Pz	0.270	3.703	Substantial evidence for H_0_
	P4	0.306	3.270	Substantial evidence for H_0_
800–1,200	F3	0.294	3.399	Substantial evidence for H_0_
	Fz	0.282	3.546	Substantial evidence for H_0_
	F4	0.275	3.633	Substantial evidence for H_0_
	C3	0.270	3.704	Substantial evidence for H_0_
	Cz	0.382	2.616	Anecdotal evidence for H_0_
	C4	0.270	3.707	Substantial evidence for H_0_
	P3	0.359	2.782	Anecdotal evidence for H_0_
	Pz	0.292	3.428	Substantial evidence for H_0_
	P4	0.347	2.886	Anecdotal evidence for H_0_

**Table 9:** Bayes factors for critical comparisons in the ERP old/new effects for new backgrounds in the item memory blocks.

Time window	Electrode	BF_10_	BF_01_	Interpretation
300–500	F3	0.271	3.691	Substantial evidence for H_0_
	Fz	0.293	3.408	Substantial evidence for H_0_
	F4	0.286	3.494	Substantial evidence for H_0_
	C3	0.423	2.364	Anecdotal evidence for H_0_
	Cz	0.310	3.227	Substantial evidence for H_0_
	C4	0.271	3.696	Substantial evidence for H_0_
	P3	0.387	2.587	Anecdotal evidence for H_0_
	Pz	0.293	3.418	Substantial evidence for H_0_
	P4	0.371	2.697	Anecdotal evidence for H_0_
500–800	F3	0.277	3.610	Substantial evidence for H_0_
	Fz	0.365	2.743	Anecdotal evidence for H_0_
	F4	0.368	2.718	Anecdotal evidence for H_0_
	C3	0.346	2.892	Anecdotal evidence for H_0_
	Cz	0.286	3.492	Substantial evidence for H_0_
	C4	0.277	3.606	Substantial evidence for H_0_
	P3	0.372	2.691	Anecdotal evidence for H_0_
	Pz	0.293	3.409	Substantial evidence for H_0_
	P4	0.461	2.171	Anecdotal evidence for H_0_
800–1,200	F3	0.299	3.349	Substantial evidence for H_0_
	Fz	0.334	2.993	Anecdotal evidence for H_0_
	F4	0.325	3.074	Substantial evidence for H_0_
	C3	0.272	3.673	Substantial evidence for H_0_
	Cz	0.270	3.698	Substantial evidence for H_0_
	C4	0.322	3.109	Substantial evidence for H_0_
	P3	0.270	3.708	Substantial evidence for H_0_
	Pz	0.272	3.677	Substantial evidence for H_0_
	P4	0.289	3.462	Substantial evidence for H_0_

The pattern was less clear for the source memory task (see Table 10). In the time windows 300–500 ms and 500–800 ms, there is only anecdotal evidence for H_0_ or H_1_ for the majority of electrodes. For electrode C4 in both time windows there is actually substantial evidence for H_1_. Old/new effects were larger for German participants relative to Chinese participants. As no significant interactions involving the source factor were found in the frequentist ERP analyzes of the source memory task in the 500 to 800 ms time interval, this is an interesting discrepancy between the results of the frequentist and the Bayesian analyzes. In the 800–1,200 ms time window, the data provide mostly substantial evidence for the H_0_.

**Table 10:** Bayes factors for critical comparisons in the ERP old/new effects in the source memory blocks.

Time window	Electrode	BF_10_	BF_01_	Interpretation
300–500	F3	0.771	1.297	Anecdotal evidence for H_0_
	Fz	1.490	0.671	Anecdotal evidence for H_1_
	F4	0.764	1.310	Anecdotal evidence for H_0_
	C3	0.597	1.674	Anecdotal evidence for H_0_
	Cz	1.783	0.561	Anecdotal evidence for H_1_
	C4	6.253	0.160	Substantial evidence for H_1_
	P3	0.587	1.703	Anecdotal evidence for H_0_
	Pz	0.783	1.277	Anecdotal evidence for H_0_
	P4	0.691	1.448	Anecdotal evidence for H_0_
500–800	F3	0.544	1.838	Anecdotal evidence for H_0_
	Fz	1.001	0.999	Anecdotal evidence for H_1_
	F4	0.702	1.424	Anecdotal evidence for H_0_
	C3	0.502	1.992	Anecdotal evidence for H_0_
	Cz	0.747	1.338	Anecdotal evidence for H_0_
	C4	4.535	0.221	Substantial evidence for H_1_
	P3	0.601	1.663	Anecdotal evidence for H_0_
	Pz	0.725	1.380	Anecdotal evidence for H_0_
	P4	0.503	1.988	Anecdotal evidence for H_0_
800–1,200	F3	0.270	3.707	Substantial evidence for H_0_
	Fz	0.291	3.433	Substantial evidence for H_0_
	F4	0.270	3.706	Substantial evidence for H_0_
	C3	0.311	3.215	Substantial evidence for H_0_
	Cz	0.270	3.707	Substantial evidence for H_0_
	C4	0.478	2.090	Anecdotal evidence for H_0_
	P3	0.281	3.557	Substantial evidence for H_0_
	Pz	0.307	3.255	Substantial evidence for H_0_
	P4	0.293	3.412	Substantial evidence for H_0_

#### 3.3.4. Summary

The ERP results revealed an old/new effect for the item memory task in both groups which was not modulated by backgrounds. Both, frequentist and Bayesian analyzes suggest that there were no cultural differences in the old/new effects. No consistent old/new effect emerged in the source memory task in the middle and late time interval. However, the Bayesian analyzes suggest that there were some cultural differences at a central recording site in one electrode in the earlier time windows.

## 4. Discussion

In this cross-cultural ERP study, we examined how changes in the scenic background affected item and source memory performance and their ERP correlates in German and Chinese young adults. Using measures corrected for response bias, we found better item memory for objects shown in front of the original background as compared to objects presented with new backgrounds. In the source memory blocks, memory performance for intact items was better than for recombined items in both cultures. However, as verified with frequentist and Bayesian analyzes and contrary to our hypotheses, neither item memory nor source memory performance for contexts were modulated by culture. The ERP results revealed an old/new effect for the item memory task in both groups which was again not modulated by backgrounds. Next, we will discuss the implications of these findings and the limitations of the current study.

### 4.1. Cultural differences in memory performance

The absence of any cultural differences of the context effect in item and source memory was unexpected given the numerous evidence for cultural differences in attention allocation outlined in the introduction (e.g., Masuda and Nisbett, 2001; Nisbett and Masuda, 2003; Chua et al., 2005). This is especially true for the item memory task, which was modeled after the seminal study by Masuda and Nisbett (2001), Experiment 2, see also Chua et al. (2005) for a similar design. Some scholars pointed out that the use of the uncorrected hit rate in the aforementioned study might have led to an overestimation of cultural differences in memory (e.g., Evans et al., 2009; Gutchess and Huff, 2016). In the present study, we analyzed both, hit rates and Pr scores. However, cultural differences in the processing of old and new backgrounds were not observed in either measure. Moreover, the results from the Bayesian t-tests suggest that our results provide evidence in favor of the null hypothesis (i.e., the absence of cultural differences). Thus, the discrepancy in the results between our study and the aforementioned studies cannot be accounted for by differences in memory assessment or quantification.

An explanation for the null results could be found in the stimulus material. We opted for a high similarity between the two objects and between the two backgrounds (see also Weigl et al., 2023). This not only facilitated counterbalancing, but also should have prevented the use of information reduction strategies (e.g., sole reliance on the color of the stimulus), which could bias memory retrieval. However, the high similarity between the objects and between the backgrounds might have made the memory task too demanding for the participants and therefore obscured cultural differences. This possibility can also be precluded as the Pr scores were well above zero (especially in the item memory task). Moreover, the values were neither close to the ceiling not to the bottom, allowing for enough variance for cultural differences to manifest. In addition, the context effect (i.e., better memory for intact than recombined pairs) was replicated (e.g., Ecker et al., 2007), but not modulated by culture, contrary to our predictions.

In a similar vein, German and Chinese participants might have differed in the perceived distinctiveness or visual saliency of the foreground object. Distinctiveness and saliency are known to affect memory-related processes (e.g., Fine and Minnery, 2009; Santangelo, 2015; Santangelo et al., 2015; Weigl et al., 2020; see also Mecklinger and Kamp, 2023, for a review). Since such cultural differences in saliency should have led to differences in memory, it seems highly unlikely that our findings are the result of differentially perceived distinctiveness or saliency. However, these stimulus properties have not been systematically controlled in this study. Future research could more thoroughly investigate how cultural differences in the processing of distinctive and salient stimuli affect item and source memory and their respective neural correlates.

Another potential reason for the absence of cultural differences could be that ORCA stimulus materials (Weigl et al., 2023) entail object-background pairings with a low semantic fit between object and background to preclude that semantic knowledge affects memory decisions. However, the lack of semantic fit might have led to similar context processing in German and Chinese participants. In support of this view, most early cross-cultural memory studies actually used material with high semantic fit (e.g., Masuda and Nisbett, 2001; Chua et al., 2005; Evans et al., 2009), suggesting that semantic relations facilitate the binding of objects and backgrounds in East Asians relative to Westerners. However, this interpretation seems implausible for the following reasons. Weigl et al. (2023) reported that the semantic fit of the object-background arrangements was rated lower by younger Chinese adults relative to younger German adults, suggesting a cultural difference in incongruency perception. In line with these results, Goto et al. (2010) reported that Asian Americans were more sensitive to incongruent object-background parings than European Americans as indexed by higher amplitudes of the N400, an ERP component sensitive to semantic congruency. They argued that East Asians process their environment to a greater extent relative to Westerners, which in turn leads to a higher context-sensitivity. This finding was replicated in other ERP studies (Goto et al., 2013; Masuda et al., 2014). Together, these results suggest that low semantic fit should have led to stronger cultural differences due to a higher sensitivity to incongruency in Chinese relative to German participants. Nevertheless, future studies could use both, congruent and incongruent object-background pairings in order to determine to what degree cultural differences in the context effect are determined by congruency.

Since neither differences in memory assessment, nor task difficulty, nor low semantic fit can account for the absence of cultural differences in our data, it seems reasonable to assume that cultural difference in context effects do not manifest in the item memory task with semantically unrelated object-background pairs. For the source memory task, however, this might not hold true as the Bayesian t-test was inconclusive.

### 4.2. Cultural differences in the ERP correlates of episodic memory

Contrary to our expectations, there was only an ERP old/new effect, which was neither modulated by backgrounds nor by culture. Moreover, no consistent old/new effect emerged in the source memory task.

The ERP old/new effect might have been delayed, because the task is very difficult and participant require more time for their recognition decision. Some evidence in favor for this position can be found in the reaction time results, which indicated that participants showed high variability in reaction times and required a great deal of time for reaching their decisions – particularly in the source memory task, in which participants of both cultures took more than 2 s to respond correctly to recombined pairs (see Table 11 for descriptive statistics). Such a “smear out” effect might also account for the fact that the old/new effects are in general rather weak or even absent.

**Table 11:** Mean and standard deviations for the mean reaction times (in ms) for correct responses in the item and source memory task.

Task	Type	Germans	Chinese
Item memory	Old item old background	1,445 ms (438 ms)	1,663 ms (655 ms)
	Old item new background	1,514 ms (434 ms)	1,737 ms (602 ms)
	New item old background	1,525 ms (430 ms)	1,788 ms (593 ms)
	New item new background	1,564 ms (451 ms)	1,828 ms (687 ms)
Source memory	Intact	1,694 ms (424 ms)	2,150 ms (826 ms)
	Recombined	2033 ms (479 ms)	2,596 ms (912 ms)

Another interpretation for the results could be that the early old/new is in fact absent. The results from the Bayesian t-test point in this direction, at least for the item memory task. Given that familiarity is not helpful for distinguishing between old and new objects due to the high similarity of the object and its distractor, the absence of an early old/new effect in the ERPs might actually be not surprising. In fact, a study by Leger and Gutchess (2021) using the mnemonic similarity task suggests that East Asians’ performance suffers more from similar distractors than Westerners’ performance. The observed old/new effects could be interpreted as the late old/new effect, which represents recollection-based retrieval. In fact, participants might have only been able to solve the task with recollection due to the high similarity of old and new objects (and backgrounds). The untypical fronto-central distribution of the latter effect could be accounted for by the use of pictorial stimuli. Consistent with this view, several recognition memory studies using similar pictorial material as the present study report broadly distributed or even frontally accentuated late old/new effects, indicative for recollection-based remembering (Gutchess et al., 2007; Höltje and Mecklinger, 2020).

For the source memory task, the absence of a significant old/new effect is more surprising. In this task, the oldness of both, object and background, made recollection a necessity for good performance. In fact, most strategies to simplify the task (e.g., only respond to the oldness of the object or relying on familiarity feeling) will not work under these circumstances.

### 4.3. Caveats and directions for future cross-cultural memory studies

Our study is associated with five main caveats. First of all, the use of an intentional study task could have been suboptimal, because participants might have selected encoding strategies, which maximize memory, rather than relying on strategies, which are preferred by the respective culture (i.e., analytic strategies by Germans and holistic strategies by Chinese). However, the intentional nature of the study task might have further been reinforced by the long instructions prior to the main experiment. We tried to prevent the use of task-specific strategies and foster the use of the culturally preferred strategies by letting the participants believe that the selection of the task was random. This approach was chosen, because pilot participants reported that they adjusted their attention allocation in response to the expected test phase. Yet, this might have led participants to simply focus on both, object and background scene, in all study phases. In a similar vein, the use of a spatial fit rating in the study task might have worked against finding cultural differences, because they led participants in both cultures to focus on both, the object and the background scene.

In conclusion, these factors (intentional task, spatial fit rating, and randomization instructions) might have induced a “holistic” processing mode in participants from both cultures. Young adults are still cognitive flexible enough to also rely on culturally non-preferred strategies (e.g., Park and Gutchess, 2006; Gutchess and Huff, 2016). For example, in an fMRI study by Goh et al. (2007), cultural differences in visual processing of objects and backgrounds were found in older adults, but not in younger adults. This high cognitive flexibility of younger adults may also have hampered finding cultural differences in the processing of contextual information. Future studies that employ incidental study tasks in order to avoid prompting participants to use culturally non-preferred strategies should be conducted to sheet light on this issue.

The second caveat is that the encoding time (2,500 ms) might have been too short to properly encode the stimuli. This might have especially hampered East Asians in forming holistic representations of the stimuli. The encoding time was chosen in order to prevent that the experiment becomes too long. As the current version of the experiment already takes 3–4 h to complete (incl. preparation), longer encoding times might have results in fatigue effects. However, performance in the memory task show that Chinese were actually better than Germans (on a trend level). This is in evidence contrary to the argument.

A third caveat is that this study was carried out under the assumption that East Asians have a more holistic processing style which promotes unitization of object and background. This in turn should lead Chinese participants to remember more on the basis of familiarity. This difference in processing was supposed to be revealed in memory performance and the ERPs. However, there is some evidence suggesting that an entity-defining framework is needed for unitization (e.g., Jäger and Mecklinger, 2009; Bader et al., 2010). An entity-defining framework means that a unit can be formed via some sort of rule (e.g., a unit can be formed with the color pink and an elephant by combining the color with the mammal). Given that our stimulus material arbitrarily arranged the objects and the backgrounds, participants might have had problems with establishing an entity-defining framework and with forming units out of these disparate materials. Thus, our material might not be suitable for promoting unitization or, together with other factors described above, might even have discouraged holistic processing. This, in turn may have worked against an effect of culture on memory. However, as discussed in section 4.1, ERP studies on the N400 suggests that East Asians in fact react stronger to semantic incongruency than Westerners (e.g., Goto et al., 2010, 2013; Masuda et al., 2014). Consequently, we would actually expect stronger cultural differences to manifest in both, the behavioral and the ERP data, due to the higher sensitivity to incongruency of East Asians relative to Westerners.

The fourth caveat is that cross-cultural studies consistently find differences between East Asians and Westerners in categorization, a process which heavily draws on semantic memory (Chiu, 1972; Ji et al., 2004; Unsworth et al., 2005). Whereas Westerners tend to classify based on taxonomy, East Asians prefer relational classifications. In an implicit memory task, Unsworth et al. (2005) found that Westerners reacted faster to categorical primes than to relational primes indicating that their semantic network is organized categorically. No differences were found for East Asians indicating that both, relational and categorical associations are stored in semantic memory. Thus, cultural differences might, at least partially, reflect differences in semantic memory. Future ERP studies, which use material allowing for categorization, might have more chances to find cultural differences in memory.

The fifth caveat is that there could be concerns about the selection of the cultures to be compared. Most studies compare Americans as representants of Westerners and Chinese or Japanese people as representants of East Asians (e.g., Masuda and Nisbett, 2001; Chua et al., 2005). However, there is evidence that Germans are less individualistic than Americans (Nisbett, 2003). The absence of cultural differences might be attributable to the selection of an unrepresentative reference sample. In a similar vein, it could be argued that the globalization in recent years might have reduced cultural differences. The same holds true for the COVID-19 pandemic. This means that German and Chinese young adults might be more similar than expected due to globalization (Nisbett, 2003) or COVID pandemic.

However, we found significant cultural differences in the expected direction in the pen test and the SCS. German participants preferred the rare pen, whereas the reverse was true for Chinese participants and German participants also had a more independent self-construal than the Chinese participants (see Weigl et al., 2023 for similar results). This suggests that our groups were representative for their respective cultures and the lack of memory by culture interactions in the main experiment cannot be attributed to sampling atypical cases or an assimilation of the cultures due to factors like globalization. Similarly, the absence of cultural effects on the ERP old/new effects cannot be attributed to a general lack of sensitivity of the ERPs to cultural differences. In support of this view, in a cross-cultural ERP study on object identification conducted in our labs with highly comparable Chinese and German groups of participants we found reliable cultural differences in the N350, an ERP measure of object model selection (Mecklinger et al., 2014). These latter results are consistent with the results of other Sino-German cross cultural ERP studies (Lewis et al., 2008; Wang et al., 2014). Taken together, this can be seen as evidence, that the behavioral and ERP data we collected in the present study were in principle sensitive enough for detecting cultural differences.

## 5. Conclusion

In contrast to some previous studies, which found cultural differences in the way changes in context affected memory (e.g., Masuda and Nisbett, 2001; Chua et al., 2005), we found no evidence for a cultural modulation of memory performance, neither in our behavioral data, nor in the ERPs. Rather, our results suggest that cultural differences in young adults do not manifest in intentional memory tasks probing memory for object-scene pairs without semantic relations when using bias-corrected memory measures. Future research should focus on elucidating under which conditions culture affects memory and under which condition memory remains unaffected.

## Data availability statement

The raw data supporting the conclusions of this article will be made available by the authors, without undue reservation.

## Ethics statement

The studies involving humans were approved by Ethic committee of the Faculty of Human and Business Sciences at Saarland University. The studies were conducted in accordance with the local legislation and institutional requirements. The participants provided their written informed consent to participate in this study.

## Author contributions

MW: conceptualization, data curation, formal analyzes, methodology, resources, supervision, writing original draft, and visualization. QS: data curation, formal analyzes, resources, methodology, and visualization. EW: data curation, formal analyzes, resources, and visualization. ZZ: conceptualization, formal analyzes, methodology, resources, supervision, and writing original draft. JL: conceptualization, funding acquisition, methodology, validation, and supervision. JK: conceptualization, funding acquisition, methodology, validation, and writing original draft. AM: conceptualization, funding acquisition, methodology, validation, supervision, and writing original draft. All authors contributed to the article and approved the submitted version.

## Funding

This work was supported by the Deutsche Forschungsgemeinschaft (DFG) Projekt ME 1588/12-1 and the Youth Innovation Promotion Association of the Chinese Academy of Sciences (2020089).

## Conflict of interest

The authors declare that the research was conducted in the absence of any commercial or financial relationships that could be construed as a potential conflict of interest.

## Publisher’s note

All claims expressed in this article are solely those of the authors and do not necessarily represent those of their affiliated organizations, or those of the publisher, the editors and the reviewers. Any product that may be evaluated in this article, or claim that may be made by its manufacturer, is not guaranteed or endorsed by the publisher.

## Footnotes

^1^Please note that the Corsi block task and the Wechsler vocabulary test described at the end of this section were performed to compare our sample with data from older adults, which were collected in a separate study. Thus, these variables will not be further explored in the present report and the results will be reported elsewhere.

^2^Please note that in the item memory task, participants only responded to the object (see section 2.3.2). Therefore, we refer to intact pairs as “old item in old scene” or “new item in new scene” in the item memory task. Similarly, recombined pairs are referred to as “old item in new scene” or “new item in old scene” in the item memory task.

^3^Due to an error in the coding of the confidence judgments, these judgments are unavailable for some conditions in some participants. We therefore refrained from analyzing these data.

^4^In case of non-significant results, Bayesian analyzes allow us to distinguish between “absence of evidence” or “evidence of absence” (van den Bergh et al., 2020).

^5^In contrast to the value of p, there are several interpretation and verbal labels for the Bayes factor. Here, we rely on the widely used labels by Jeffreys (1961): BF < 3 is interpreted as “anecdotal evidence,” 3 < BF < 10 as “substantial evidence,” and BF > 10 as “strong evidence.”
